# Accuracy of cytokeratin 18 (M30 and M65) in detecting non-alcoholic steatohepatitis and fibrosis: A systematic review and meta-analysis

**DOI:** 10.1371/journal.pone.0238717

**Published:** 2020-09-11

**Authors:** Jenny Lee, Yasaman Vali, Jérôme Boursier, Kevin Duffin, Joanne Verheij, M. Julia Brosnan, Koos Zwinderman, Quentin M. Anstee, Patrick M. Bossuyt, Mohammad Hadi Zafarmand

**Affiliations:** 1 Epidemiology and Data Science, Amsterdam UMC, University of Amsterdam, Amsterdam, The Netherlands; 2 Hepato-Gastroenterology Department, Angers University Hospital, Angers, France; 3 HIFIH Laboratory, UPRES EA3859, Angers University, Angers, France; 4 Lilly Research Laboratories, Eli Lilly and Company Ltd (LLY), Indianapolis, IN, United States of America; 5 Department of Pathology, Amsterdam UMC, University of Amsterdam, Amsterdam, The Netherlands; 6 Internal Medicine Research Unit, Pfizer Inc, Cambridge, MA, United States of America; 7 The Newcastle Liver Research Group, Translational & Clinical Research Institute, Faculty of Medical Sciences, Newcastle University, Newcastle upon Tyne, United Kingdom; 8 Newcastle NIHR Biomedical Research Centre, Newcastle upon Tyne Hospitals NHS Foundation Trust, Newcastle upon Tyne, United Kingdom; Medizinische Fakultat der RWTH Aachen, GERMANY

## Abstract

**Introduction:**

Association between elevated cytokeratin 18 (CK-18) levels and hepatocyte death has made circulating CK-18 a candidate biomarker to differentiate non-alcoholic fatty liver from non-alcoholic steatohepatitis (NASH). Yet studies produced variable diagnostic performance. We aimed to provide summary estimates with increased precision for the accuracy of CK-18 (M30, M65) in detecting NASH and fibrosis among non-alcoholic fatty liver disease (NAFLD) adults.

**Methods:**

We searched five databases to retrieve studies evaluating CK-18 against a liver biopsy in NAFLD adults. Reference screening, data extraction and quality assessment (QUADAS-2) were independently conducted by two authors. Meta-analyses were performed for five groups based on the CK-18 antigens and target conditions, using one of two methods: linear mixed-effects multiple thresholds model or bivariate logit-normal random-effects model.

**Results:**

We included 41 studies, with data on 5,815 participants. A wide range of disease prevalence was observed. No study reported a pre-defined cut-off. Thirty of 41 studies provided sufficient data for inclusion in any of the meta-analyses. Summary AUC [95% CI] were: 0.75 [0.69–0.82] (M30) and 0.82 [0.69–0.91] (M65) for NASH; 0.73 [0.57–0.85] (M30) for fibrotic NASH; 0.68 (M30) for significant (F2-4) fibrosis; and 0.75 (M30) for advanced (F3-4) fibrosis. Thirteen studies used CK-18 as a component of a multimarker model.

**Conclusions:**

For M30 we found lower diagnostic accuracy to detect NASH compared to previous meta-analyses, indicating a limited ability to act as a stand-alone test, with better performance for M65. Additional external validation studies are needed to obtain credible estimates of the diagnostic accuracy of multimarker models.

## Introduction

Non-alcoholic fatty liver disease (NAFLD), a condition with a complex and multifactorial etiology, has rapidly emerged as the most common cause of chronic liver disease in the United States and Europe [1, 2]. The global prevalence is approximately 25%, representing a wide histological spectrum from simple steatosis (NAFL), non-alcoholic steatohepatitis (NASH) [3] to hepatic fibrosis. Fibrosis is the strongest predictor for long-term clinical outcomes in NAFLD patients, thereby, a key target event for patient stratification and clinical trial recruitment [4].

The clinical reference standard for detecting NASH activity and fibrosis stages is a liver biopsy, a practice with well-established limitations [5–7]. As such, only patients at highest risk should be pre-selected for such an invasive and resource intensive procedure. The discovery of less invasive methods with performance comparable to liver biopsy has become essential.

Several blood-based biomarkers have been studied for their ability to identify NASH or fibrosis. Cytokeratin 18 (CK-18) is the main intermediate filament protein in hepatocytes and is released upon the initiation of cell death. The association between elevated CK-18 levels and cell death in the liver [8, 9] has made circulating CK-18 (both M30 and M65 antigens) a candidate marker for detecting NASH and fibrosis [10], as a stand-alone test and, more recently, as part of multimarker models.

Although the M30 and M65 antigens are of the same protein, there is a mechanistic distinction between the two. M30 measures the caspase-cleaved CK-18 revealed during apoptosis, while M65 measures the full-length protein, including both caspase-cleaved and intact CK-18, which is released from cells undergoing necrosis [11].

In recommendations by the EASL-EASD-EASO Clinical Practice Guidelines [12] the performance of CK-18 M30 to differentiate NASH from NAFL was judged modest, as per data from a meta-analysis of 11 studies [13]. The Asia-Pacific Working Party on NAFLD [14] similarly concluded modest performance, referencing a meta-analysis of 10 studies [15]. A single study mentioned in both guidelines criticized CK-18 for its limited performance for detecting NASH at a threshold of 165 U/L [10]. However, it is not clear what thresholds would then maximize the test’s sensitivity or specificity.

We found several limitations and methodological concerns in the above-mentioned meta-analyses. One performed a meta-analysis on only the M30 antigen in detecting NASH, with the rationale that M65 performed similarly [13]. However, it has been shown that M65 outperforms M30 [9]. Further, we found several methodological concerns in the systematic review by Chen et al. such as overlapping patient populations included in the meta-analysis [15].

An updated and more methodologically robust meta-analysis would be able to generate, in principle, summary estimates with increased precision and more general validity. To address this need, we aimed to conduct a systematic review and meta-analysis of the accuracy of both CK-18 antigens (M30 and M65) in identifying NASH, fibrotic NASH, and fibrosis stages among NAFLD adults.

## Materials and methods

This systematic review was conducted as part of the evidence synthesis efforts of the LITMUS (Liver Investigation: Testing Marker Utility in Steatohepatitis) project, funded the European Union’s IMI2, aiming to evaluate biomarkers for use in NAFLD. The protocol of the complete systematic review is available in PROSPERO (registration number: CRD42018106821). This study report was prepared using the PRISMA-DTA statement, see PRISMA checklist in S1 Table in S1 File.

### Search strategy

A comprehensive search strategy, containing words in the title/abstract or text words across the record and the medical subject heading (MeSH), was developed with a search specialist. MEDLINE (via OVID), EMBASE (via OVID), PubMed, Science Citation Index, and CENTRAL (The Cochrane Library) were searched to retrieve potentially eligible studies from inception to August 2018 (see S2 Table in S1 File). We further conducted a manual screening of relevant systematic reviews and reference lists and contacted partners within the LITMUS consortium. The search was updated in May 2019, and again in June 2020.

### Study selection

Search results of all databases were merged and deduplicated using Endnote. Titles were screened by one reviewer (YV); a second reviewer independently screened 10% (MHZ). Abstract and full text screening was conducted by two independent reviewers (JL and YV), following pre-established inclusion and exclusion criteria. Any discrepancies were resolved by discussion between the two reviewers. Title and abstract screening phases were conducted on Rayyan QCRI (https://rayyan.qcri.org).

### Inclusion and exclusion criteria

We searched for studies including adults (≥18 years) with clinical suspicion or biopsy proven NAFLD, with paired data on liver histology and CK-18 (M30 or M65). Diagnostic accuracy studies reported in full articles in peer-reviewed journals, or as conference abstracts, in any language were eligible. Studies with insufficient information for making decisions on inclusion, for evaluating methodological quality, or for calculating diagnostic accuracy were excluded. Study groups with a mix of conditions (e.g. viral hepatitis) were only included if outcomes were separately reported for NAFLD patients.

The target conditions for this systematic review were NASH, fibrotic NASH, and liver fibrosis. The NAFLD Activity Score (NAS) [16] is the most commonly used pathologic criterion for evaluating NASH. We considered a threshold value of NAS ≥4 with at least one point for each criteria of steatohepatitis for the characterization of NASH. See S3 Table in S1 File for different histological scoring systems developed to characterize NAFLD progression. Fibrotic NASH was defined using the above-mentioned criteria for NASH and at least F1 or more.

A five-point scoring system (F0-F4), developed by the NASH clinical research network (NASH CRN) [17], is the most commonly used for fibrosis staging. Studies assessing significant (≥F2) and advanced (≥F3) fibrosis were included. See S4 Table in S1 File for different scoring systems for liver fibrosis, and S5 Table in S1 File for a conversion grid of the different scoring systems.

### Data extraction and quality assessment

The following information was extracted: study characteristics, clinical characteristics, index test features, liver biopsy features, and data that allowed construction of a 2x2 contingency table (true positives, true negatives, false positive and false negatives) to assess the performance of the index test. For studies that reported accuracy data for multiple thresholds, all data were extracted.

When pertinent data were not reported, the corresponding study author was contacted. Data were extracted independently and cross-checked by two reviewers (JL and YV).

The Quality Assessment of Diagnostic Accuracy Studies (QUADAS-2) tool [18] was used to assess methodological quality of all available full text studies. Two reviewers (JL and YV) independently evaluated the risk of bias and concerns about applicability of the included primary studies using the four domains of QUADAS-2, assigning each study with a judgement of ‘low’, ‘high’, or ‘unclear’ risk.

### Statistical analysis

Included studies were classified into five groups for meta-analysis, based on the availability of data on the CK-18 antigens and target conditions: (1) CK-18 M30 for detecting NASH, (2) CK-18 M65 for detecting NASH, (3) CK-18 M30 for detecting fibrotic NASH, (4) CK-18 M30 for detecting significant fibrosis, and (5) CK-18 M30 for detecting advanced fibrosis.

Sensitivity and specificity estimates from each study, with respective 95% confidence intervals (95% CI), were graphically illustrated as forest plots, for each reported threshold, using RevMan.

Two different meta-analytical methods were applied for the combinations of CK-18 antigens and target conditions based on the number of reported threshold values. For groups 1–3, we applied a linear mixed effects multiple thresholds model (diagmeta package in R) as a majority of the primary studies reported multiple threshold values. The multiple thresholds model utilizes the number of true and false positives and true and false negatives at every threshold to produce summary receiver operating characteristic (SROC) curves. With the model, we could calculate estimates of sensitivity, specificity at any given threshold. We calculated the threshold value that would maximize Youden's J statistic (also called Youden's index): the sum of sensitivity and specificity minus 1.

We computed estimates of positive and negative predictive values in settings with different disease prevalence. We further assessed thresholds of the index test required to achieve pre-specified high values of sensitivity and specificity. The minimally acceptable performance levels of AUC and sensitivity and specificity for the index test was 0.80, for it to exceed that of other NAFLD-related screening and diagnostic biomarkers.

As a majority of the primary studies in groups 4 and 5 reported only a single threshold value, we applied a bivariate logit-normal random-effects model (mada package in R) to compute summary estimates of sensitivity and specificity. SROC curves were constructed to represent the overall diagnostic accuracy of the index test.

Publication bias was not formally evaluated as no accepted statistical tests can reliably discriminate publication bias from other sources of bias in diagnostic meta-analyses [19]. Heterogeneity between and within studies was incorporated by calculating 95% prediction intervals [20]. The confidence interval around the summery point reflect the statistical imprecision around the mean. The prediction region around the summary point indicates the region where we would expect results from a new study in the future to lie. It reflects both the uncertainty around the mean and the between study heterogeneity and is therefore wider than the confidence region.

We investigated the influence of studies with compromised methodological quality by excluding those at high risk of bias or with applicability concerns in a sensitivity analysis. We further evaluated the effect of pooling data from various ELISA assays by excluding studies that either did not disclose the assay used or used one from a manufacturer that was not PEVIVA. Sensitivity analysis was also conducted among solely biopsy-proven NAFLD patients, excluding those with clinically suspected NAFLD.

All analyses were conducted using R for Windows (Version 3.6.0; R Foundation for Statistical Computing, Vienna, Austria).

## Results

### Search results

**Fig 1:**
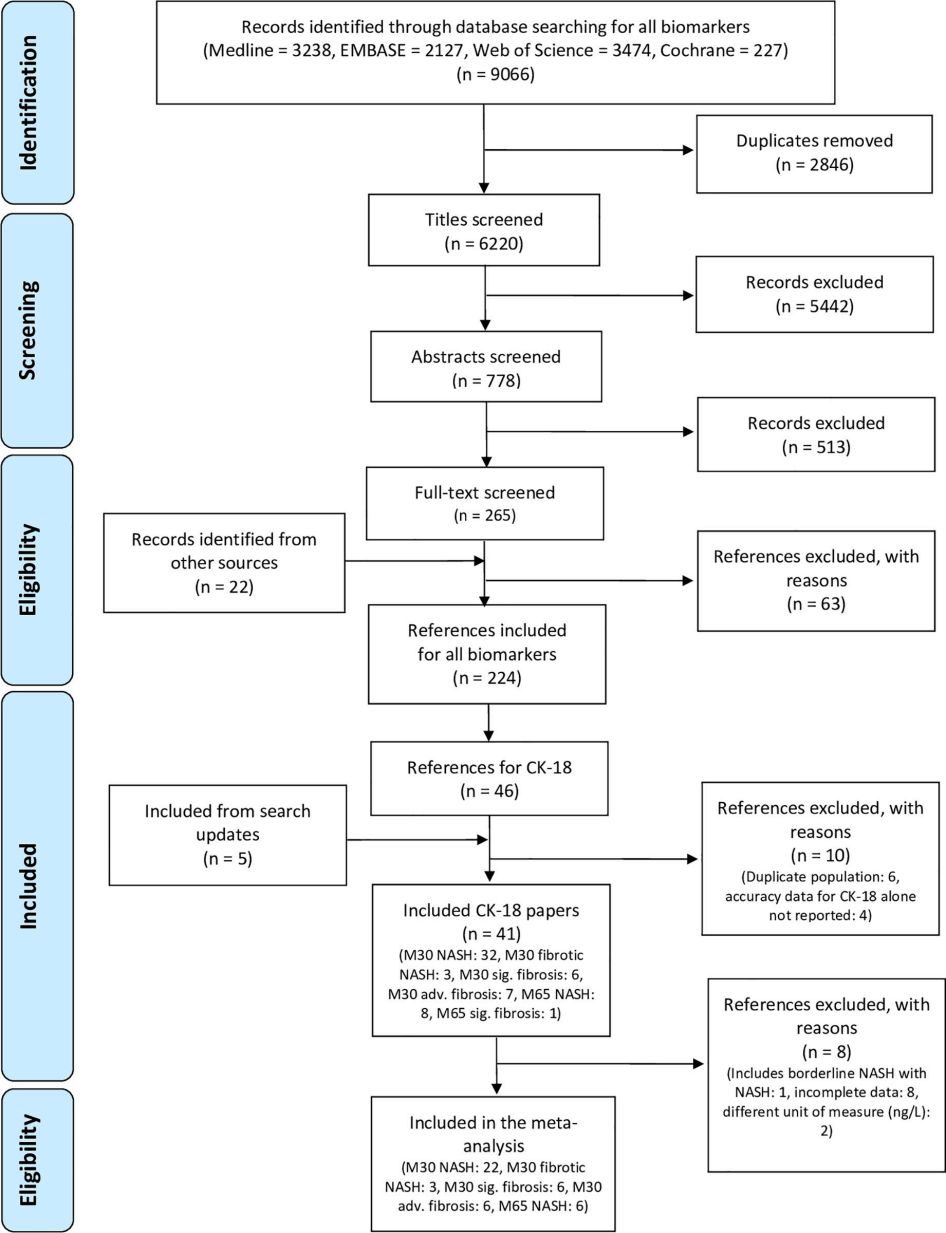
PRISMA flow diagram of included primary studies.

Our initial search of all biomarkers identified 6,220 studies post deduplication. Following the pre-defined inclusion and exclusion criteria, 778 studies were eligible for abstract screening, of which 265 underwent full-text review. A total of 46 study reports were included for CK-18. Following the exclusion of 10 and inclusion of five studies from the two search updates, a total of 41 studies (5,815 participants) could be included in the present systematic review (Fig 1). Thirty studies were included in one or more of the meta-analyses.

### Study characteristics

Characteristics of the included studies can be found in Table 1. A majority of the studies (32/41) had included NAFLD patients with mean BMI <35. A relatively wide range of disease prevalence was observed; 21% to 85% for NASH, 21% to 62% for fibrotic NASH, 18% to 59% for significant fibrosis and 19% to 36% for advanced fibrosis. The publication year spanned from 2006 to 2020; 27 studies were published after 2012.

**Table 1:** Characteristics of all included studies.

Study ID	Country	*N* (female)	Target condition	Prev (%)	Population	BMI, mean (SD)	ALT, median (IQR)	AST, median (IQR)	Comorbidities (%)
1. Aida 2014 [21]	Japan	116 (75)	NASH	44	Biopsy proven NAFLD	27.2 (18.8–45.9)^†^	52 (31–266)	42 (13–256)	NR
2. Cao 2013 [22]	China	95 (73)	NASH	46	Biopsy proven or clinical suspicion of NAFLD	28.5 (2.8)	57.0 (48.0–71.0)	48.0 (44.0–54.0)	DM: 24 HTN: 48 DL: 86
3. Chan 2014 [23]	Malaysia	93 (48)	NASH	42	US diagnosed NAFLD	29.4 (3.8)	70 (44–109)	41 (28–64)	DM: 59 HTN: 88 DL: 97
4. Chuah 2019 [24]	Malaysia	196 (99)	Fibrotic NASH	21	US diagnosed NAFLD	29.8 (4.5)	67 (44‐105)	39 (29‐61)	T2DM: 46 HTN: 58 DL: 80 Obesity: 86
5. Cusi 2013 [10]	USA	318 (113)	NASH	63	Obese patients with biopsy proven NAFLD	33.3 (0.9)	40 (1)^‡^	55 (2) ^‡^	NR
6. Darweesh 2019 [25]	Egypt	25 (55.6)	Steatosis	NR	Biopsy proven NAFLD	33.52 (4.56)	50.57 (31.06)	48.29 (46.51)	NR
7. Dvorak 2014 [26]	Czech Republic	56 (NR)	NASH	68	Biopsy proven NAFLD	29.6 (4.3)	120 (90) ^‡^	66 (60) ^‡^	NR
8. Ergelen 2015 [27]	Turkey	87 (44)	Sig. fibrosis	39	Biopsy proven NAFLD	30.6 (5.4)	77.8 (56.1) ^‡^	49.6 (30.5) ^‡^	NR
			Adv. fibrosis	22					
9. Feldstein 2009 [8]	USA	139 (88)	NASH	50	Biopsy proven NAFLD	34.2 (30.3–37.8) ^†^	43.0 (31.0–62.0)	66.0 (46.0–109.0)	DM: 19 HTN: 43 HL: 60
10. Grigorescu 2012 [28]	NR	79 (23)	NASH	75	Biopsy proven NAFLD	30 (3.8)	76.8 (39.3) ^‡^	35.9	T2DM: 16 HTN: 19
11. Hasegawa 2015 [29]	Japan	41 (7)	NASH	49	US and CT diagnosed NAFLD	NR	75.3 (68.4) ^‡^	53.6 (46.8) ^‡^	NR
12. Huang 2017 [30]	Taiwan	76 (22)	Sig. fibrosis	18	Biopsy proven NAFLD	28.7 (4.4)	117 (87.9) ^‡^	63.1 (33.3) ^‡^	DM: 54 HTN: 65
			Adv. fibrosis	9					
13. Joka 2011 [9]	Germany	22 (7)	NASH	55	Biopsy proven NAFLD	27 (1)	75.5 (9.5) ^‡^	NR	NR
14. Kamada 2013 [31]	Japan	126 (56)	NASH	85	Biopsy proven NAFLD	27.5 (5.1)	95.8 (72.0)	62.9 (39.3)	NR
15. Kawanka 2015 [32]	Japan	146 (78)	NASH	71	Biopsy proven NAFLD	26.8	61 (12–264)	38 (14–204)	NR
16. Kazankov 2016 [33]	Australia, Italy	331 (112)	NASH	40	Biopsy proven NAFLD	29.2 (4.5)	67.6	40.7	DM: 20
17. Kim 2013 [34]	Korea	108 (35)	NASH	62	Biopsy proven NAFLD	28.71 (3.77)	108.68 (82.07) ^‡^	63.54 (41.62) ^‡^^†^	MetS: 48
18. Kobayashi 2017 [35]	Japan	229 (107)	NASH Fibrotic NASH	61 45	Biopsy proven NAFLD	26.6	79.2	50.7	DM: 45 HTN: 42 DL: 56
19. Liu 2016 [36]	China	48 (13)	NASH	65	Biopsy proven NAFLD	26.9 (0.5)	68.7 (7.4) ^‡^	NR	NR
20. Liu 2019 [37]	China	82 (23.5)	NASH	47	Biopsy proven NAFLD	26.8 (3.3)	80.5 (76.4)	47.9 (31.8)	DM: 32 HTN: 35
21. Malik 2009 [38]	USA	95 (37)	NASH	63	Biopsy proven NAFLD	31.3 (4.2)	74.5 (9.7) ^‡^	NR	T2DM: 27 HTN: 49
22. Mohammed 2019 [39]	Egypt	62 (62)	NASH	66	US proven NAFLD	30.8 (4.02)	75.53 (22.3)	69 (29.5)	MetS: 59
23. Musso 2010 [40]	NR	40 (12)	NASH	58	Biopsy proven NAFLD	25.1 (1.6)	120.7 (8) ^‡^	48 (3) ^‡^	MetS: 43
24. Papatheodoridis 2010 [41]	Greece	58 (26)	NASH	52	Biopsy proven NAFLD	28.6 (4.5)	75.4	39.5	DM: 16
25. Pimentel 2016 [42]	USA	183 (73)	NASH Adv. fibrosis	49 19	Biopsy proven NAFLD	34 (7)	50.6 (32) ^‡^	75.8 (50) ^‡^	T2DM: 36 HTN: 52
26. Rosso 2016 [43]	Italy	105 (29)	Sig. fibrosis	59	Biopsy proven NAFLD	28.1 (3.9)	65 (57–79)	36 (33–41)	NR
			Adv. fibrosis	36					
27. Shen 2012 [44]	China	147 (65)	NASH	47	Biopsy proven NAFLD	27.4 (3.9)	73 (45) ^‡^	NR	T2DM: 48 HTN: 43
28. Tada 2018 [45]	Japan	170 (91)	NASH	76	Biopsy proven NAFLD	27.6 (24.9–30.7) ^†^	79 (49–126)	52 (35–82)	DM: 51 HTN: 28 DL: 44
29. Tamimi 2011 [46]	USA	95 (47)	NASH	43	Clinically suspected NASH	31.4 (5.1)	53.5 (32–87)	54 (38–75)	DM: 27 HTN: 45 MetS: 50 HL: 53
30. Valva 2018 [47]	Argentina	34 (15)	Sig. fibrosis	18	Biopsy proven NAFLD	NR	81.5 (31–279)	52.5 (22–208)	Obesity: 25
31. Wieckowska 2006 [48]	USA	39 (21)	NASH	31	Biopsy proven NAFLD	31.5 (4.0)	73.0 (54.0–104.0)	58.0 (46.0–76.0)	DM: 31 HTN: 46 HL: 46
32. Yang 2015 [49]	China	179 (93)	NASH	38	Biopsy proven NAFLD	NR	116 (30.2) ^‡^	60 (22.1) ^‡^	NR
33. Yilmaz 2007 [50]	Turkey	83 (38)	Sig. fibrosis	20	Suspected NAFLD	30.3 (4.8)	60 (10–184)	42 (16–102)	DM: 15 HTN: 34 MetS: 35
34. Younes 2018 [51]	Italy	292 (91)	NASH Adv. fibrosis	77 25	Biopsy proven NAFLD	28.9 (4.1)	66 (61–71)	36 (35–38)	MetS: 32 DM: 20
35. Younossi 2008 [52]	USA	69 (46)	NASH	32	Biopsy proven NAFLD	NR	27.1 (18.4) ^‡^	36.6 (27.3) ^‡^	NR
36. Zheng 2020 [53]	China	38 (36.2)	NASH	53	Biopsy proven NAFLD (ALT ≤ 35 (men), ≤ 23 (women))	26.05 (3.33)	27.70 (7.77)	25.77 (6.75)	DM: 36 MetS: 55 HTN: 35
37. Anty 2010 [54]	France	310 (267)	NASH	NR	Morbidly obese, bariatric surgery patients	44.7 (5.5)	35.3 (35.7) ^‡^	NR	NAS<5 DM: 19.6 MetS: 47.6 NAS≥5 DM: 43.6 MetS: 82.1
38. Boursier 2018 [55]	France, Belgium	846 (525)	NASH Fibrotic NASH	54 23	Biopsy proven NAFLD, obese patients, morbidly obese patients referred for bariatric surgery	38.5 (7.6)	49.7 (31.7) ^‡^	35.5 (19.6) ^‡^	MetS: 68 DM: 27
			Sig. fibrosis	51					
			Adv. Fibrosis	17					
39. Diab 2008 [56]	USA	55 (68)	NASH	40	Bariatric surgery patients	48 (43–54) ^†^	23.0 (18.0–29.0)	21.5 (16.0–33.0)	DM: 41 HTN: 67 DL: 57
40. Pirvulescu 2012 [57]	Romania	59 (42)	NASH (incl. borderline NASH)	22	Overweight, obese and morbidly obese patients referred for bariatric surgery	47.3 (8.1)	37.8 (13.6)^‡^	29.3 (10.1) ^‡^	NR
41. Younossi 2011 [58]	USA	79 (61)	NASH	51	Biopsy proven NAFLD	47.56 (8.07)	36.44 (28.05) ^‡^	27.22 (19.39) ^‡^	DM: 24

^†^ Median and interquartile range.

^‡^ Mean and standard deviation.

NR: not reported, DM: diabetes mellitus, T2DM: type 2 diabetes mellitus, HTN: hypertension, DL: dyslipidemia, MetS: metabolic syndrome, US: ultrasound, CT: computerized tomography scan.

Thirty-two studies investigated the accuracy of M30 in detecting NASH, and three for fibrotic NASH. The accuracy of M30 in detecting significant and advanced fibrosis was studied in six and seven studies, respectively. We further identified eight diagnostic accuracy studies of M65 for NASH and one study of M65 for significant fibrosis.

### Quality assessment

The methodological quality of the 41 studies, assessed with QUADAS-2, is summarized in S1 and S2 Figs in S1 File. Ten studies were scored as high risk of bias in the patient selection domain [9, 10, 29, 31, 36, 37, 40, 47, 50, 58]. No study had low risk of bias in the index test domain, with 22 judged as high risk, due to the lack of a pre-established threshold value for CK-18.

Seven studies were scored as unclear risk of bias in the reference standard domain, for failing to report whether biopsy reviewers were blinded to clinical data [8, 9, 29, 32, 40, 49, 57]. Only three studies were classified as at high risk of bias for flow and timing [10, 25, 40]. We further graded four studies with high concern regarding applicability in the patient selection domain [30, 46, 54, 57].

### NASH

#### Accuracy of CK-18 M30 in detecting NASH

**Fig 2:**
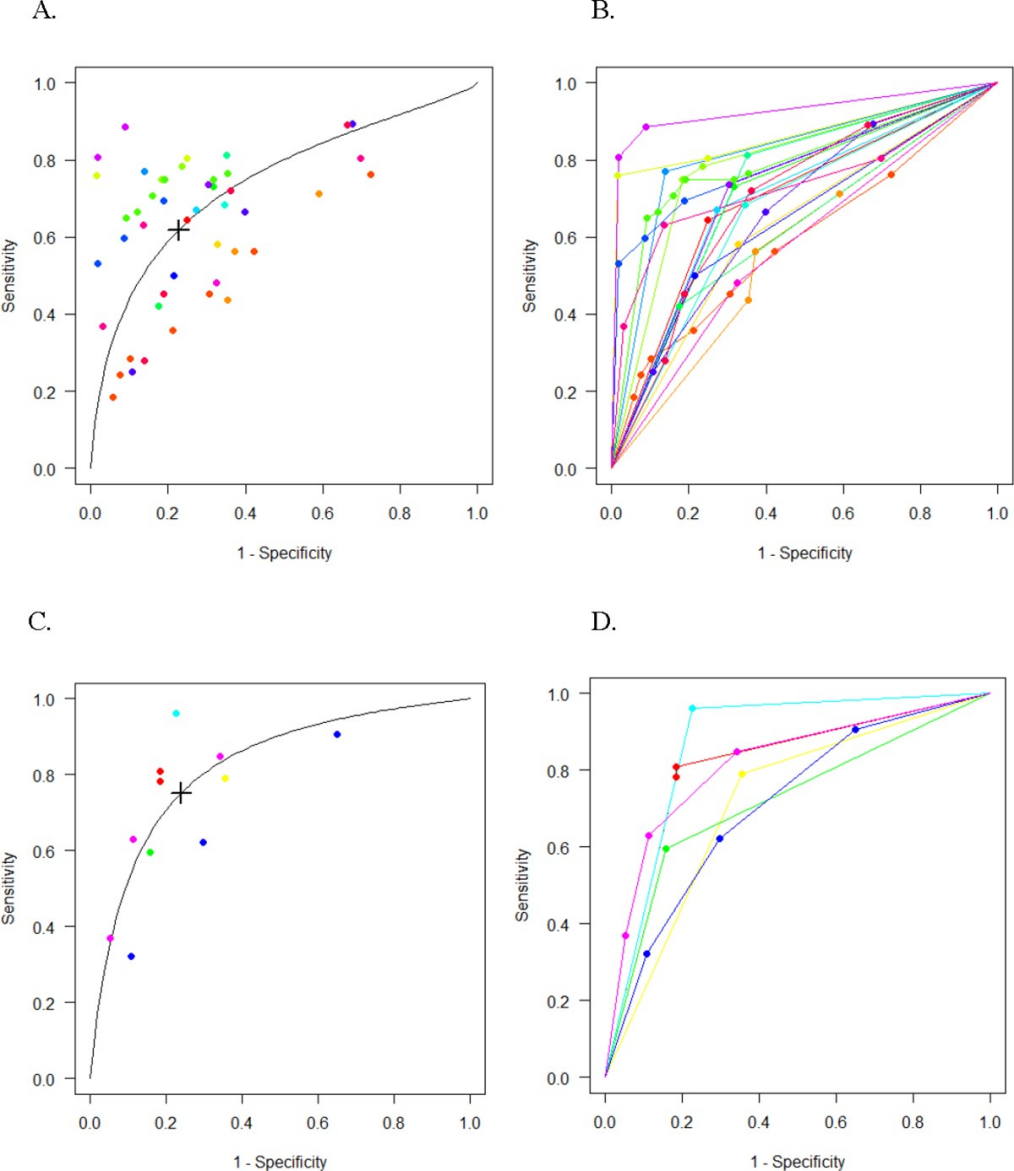
Multiple threshold SROC and ROC curves for detecting NASH. Multiple threshold SROC and ROC curves for CK-18 M30 (A-B) and M65 (C-D) in detecting NASH. Each point represents a reported threshold value, points of the same color represent thresholds reported within the same study. The x-axis indicates 1 –specificity, and the y-axis, sensitivity. The cross in the SROC curve indicates the Youden-based threshold value: A. Youden-threshold: 304 U/L, sensitivity: 0.61 (95% CI: 0.51–0.71), specificity: 0.81 (95% CI: 0.71–0.88), AUC: 0.75 (95% CI: 0.69–0.82) for CK-18 M30. C. Youden-threshold: 478 U/L, sensitivity: 0.75 (95% CI: 0.51–0.90), specificity: 0.76 (95% CI: 0.49–0.91), AUC: 0.82 (95% CI: 0.69–0.91) for CK-18 M65.

A total of 22 studies (3,503 participants, 2,010 with NASH) were included in the meta-analysis of the diagnostic accuracy of M30 in detecting NASH (S3 Fig in S1 File). Ten studies reported multiple threshold values, resulting in 47 thresholds (41 unique values) included in our model. The thresholds spanned from 111 to 670 U/L. The multiple thresholds model produced a summary area under the receiver operating characteristic curve (AUC) of 0.75 (95% CI: 0.69–0.82) with a mean sensitivity of 0.61 (95% CI: 0.51–0.71) and mean specificity of 0.81 (95% CI: 0.71–0.88). The Youden-threshold value was 304 U/L (Fig 2A).

Using the multiple thresholds model, we calculated the positive predictive value (PPV) and the negative predictive value (NPV) under different clinical settings (5% to 70% NASH prevalence) for desired levels of sensitivity and specificity (Table 2). Optimizing sensitivity (0.80 to 0.90), we found corresponding specificity values, ranging from 0.51 to 0.23 at threshold values 127 to 191 U/L (Table 2). High NPV (0.91–0.96) values were observed at lower prevalence setting of 10% and 20%. The corresponding PPV ranged from 0.12 to 0.29.

**Table 2:** Performance of M30 in detecting NASH: Positive and negative predictive values for different settings of NASH prevalence.

A. Fixed sensitivity values (0.80, 0.85, 0.90)															
**Prev**	**Fixed 0.80 sensitivity**					**Fixed 0.85 sensitivity**					**Fixed 0.90 sensitivity**				
	**Cut-off**	**Sp**	**PPV**	**NPV**	**Mis%**	**Cut-off**	**Sp**	**PPV**	**NPV**	**Mis%**	**Cut-off**	**Sp**	**PPV**	**NPV**	**Mis%**
**0.05**	191	0.51	0.08	0.98	48	161	0.38	0.07	0.98	59	127	0.23	0.06	0.98	72
**0.10**			0.15	0.96	46			0.13	0.96	56			0.12	0.96	69
**0.20**			0.29	0.91	43			0.26	0.91	52			0.23	0.91	62
**0.30**			0.41	0.85	40			0.37	0.86	47			0.34	0.86	56
**0.40**			0.52	0.79	37			0.48	0.79	43			0.45	0.79	49
**0.50**			0.62	0.72	35			0.58	0.72	38			0.55	0.72	43
**0.70**			0.79	0.52	29			0.76	0.52	29			0.74	0.52	30
B. Fixed specificity values (0.80, 0.85, 0.90)															
**Prev**	**Fixed 0.80 specificity**					**Fixed 0.85 specificity**					**Fixed 0.90 specificity**				
	**Cut-off**	**Se**	**PPV**	**NPV**	**Mis%**	**Cut-off**	**Se**	**PPV**	**NPV**	**Mis%**	**Cut-off**	**Se**	**PPV**	**NPV**	**Mis%**
**0.05**	304	0.61	0.14	0.98	21	340	0.56	0.17	0.97	17	399	0.48	0.21	0.97	12
**0.10**			0.25	0.95	22			0.28	0.94	18			0.33	0.94	15
**0.20**			0.42	0.89	24			0.47	0.88	21			0.52	0.87	19
**0.30**			0.56	0.82	26			0.60	0.81	25			0.65	0.79	24
**0.40**			0.66	0.75	28			0.70	0.73	28			0.75	0.71	28
**0.50**			0.75	0.66	31			0.78	0.64	31			0.82	0.62	33
**0.70**			0.87	0.46	35			0.89	0.43	37			0.91	0.41	42

Prev: prevalence, Sp: specificity, Se: sensitivity, PPV: positive predictive value, NPV: negative predictive value, Mis%: percent misclassified.

**Fig 3:**
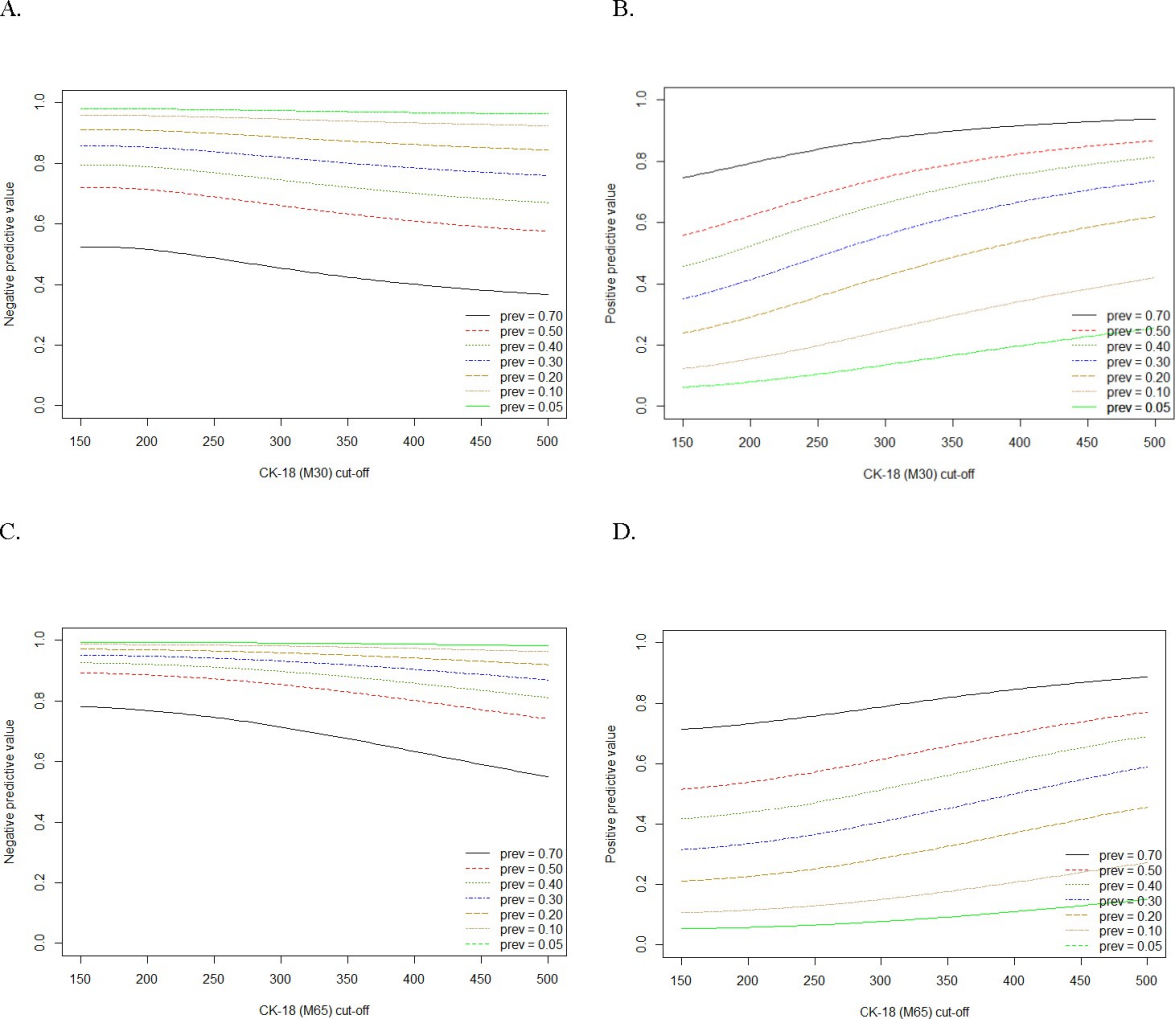
Positive and negative predictive values and thresholds for detecting NASH. Plots illustrating the negative and positive predictive values of M30 (A-B) and M65 (C-D) in detecting NASH at corresponding threshold values, projected by the multiple thresholds model. Each colored line represents a different prevalence setting, ranging from 5% to 70%. The y-axis indicates the predictive value and the x-axis indicated the threshold values for CK-18.

When fixing specificity values (0.80 to 0.90), the corresponding sensitivity ranged from 0.48 to 0.61 (Table 2) with threshold values between 304 and 399 U/L. High NPV (0.87 to 0.95) were again seen for low prevalence settings (10 to 20%). A graphical representation of the predictive values in different prevalence settings can be seen in Fig 3A and 3B.

#### Accuracy of CK-18 M65 in detecting NASH

In the meta-analysis of M65 in detecting NASH, we analyzed six studies with a total of 414 participants (220 with NASH) (S4 Fig in S1 File). Eleven unique threshold values were included in the model, ranging from 340 to 1183 U/L. The combined AUC was 0.82 (95% CI: 0.69–0.91) with a mean sensitivity of 0.75 (95% CI: 0.51–0.90) and mean specificity of 0.76 (95% CI: 0.49–0.91) at Youden-threshold of 478 U/L (Fig 2C).

We again investigated the PPV and NPV in various clinical settings (Table 3, Fig 3C and 3D). Fixing sensitivity from 0.80 to 0.90, the specificity ranged from 0.70 to 0.51 at threshold values of 337 to 437 U/L (Table 3). NPV in lower prevalence settings (10–20%) ranged from 0.93 to 0.98 with corresponding PPV from 0.17 to 0.40. Similar patterns were observed for optimizing specificity over sensitivity (Table 3). Within a NASH prevalence of 10% or 20% we found PPV and NPV ranged from 0.28 to 0.56 and from 0.88 to 0.96, respectively.

**Table 3:** Performance of M65 in detecting NASH: Positive and negative predictive values for different settings of NASH prevalence.

A. Fixed sensitivity values (0.80, 0.85, 0.90)															
**Prev**	**Fixed 0.80 sensitivity**					**Fixed 0.85 sensitivity**					**Fixed 0.90 sensitivity**				
	**Cut-off**	**Sp**	**PPV**	**NPV**	**Mis%**	**Cut-off**	**Sp**	**PPV**	**NPV**	**Mis%**	**Cut-off**	**Sp**	**PPV**	**NPV**	**Mis%**
**0.05**	437	0.70	0.13	0.99	30	391	0.63	0.11	0.99	36	337	0.51	0.09	0.99	47
**0.10**			0.23	0.97	29			0.20	0.97	35			0.17	0.98	45
**0.20**			0.40	0.93	28			0.36	0.94	33			0.32	0.95	41
**0.30**			0.54	0.89	27			0.49	0.91	30			0.44	0.95	37
**0.40**			0.64	0.84	26			0.60	0.86	28			0.55	0.88	33
**0.50**			0.73	0.78	25			0.69	0.81	26			0.65	0.84	30
**0.70**			0.86	0.60	23			0.84	0.64	22			0.81	0.67	22
B. Fixed specificity values (0.80, 0.85, 0.90)															
**Prev**	**Fixed 0.80 specificity**					**Fixed 0.85 specificity**					**Fixed 0.90 specificity**				
	**Cut-off**	**Se**	**PPV**	**NPV**	**Mis%**	**Cut-off**	**Se**	**PPV**	**NPV**	**Mis%**	**Cut-off**	**Se**	**PPV**	**NPV**	**Mis%**
**0.05**	515	0.71	0.16	0.98	20	575	0.63	0.18	0.98	16	665	0.52	0.22	0.97	12
**0.10**			0.28	0.96	21			0.32	0.95	17			0.37	0.94	14
**0.20**			0.47	0.92	22			0.51	0.90	19			0.56	0.88	18
**0.30**			0.60	0.86	23			0.64	0.84	22			0.69	0.81	21
**0.40**			0.70	0.80	24			0.74	0.76	24			0.78	0.74	25
**0.50**			0.79	0.73	25			0.81	0.70	26			0.84	0.65	29
**0.70**			0.89	0.53	26			0.91	0.50	30			092	0.45	37

Prev: prevalence, Sp: specificity, Se: sensitivity, PPV: positive predictive value, NPV: negative predictive value, Mis%: percent misclassified.

#### Accuracy of CK-18 M30 in detecting fibrotic NASH

Three studies provided sufficient data for analysis of M30 in detecting fibrotic NASH, with a combined total of 1,271 participants (343 with fibrotic NASH) (S5 Fig in S1 File). Two studies investigated M30 as part of a multimarker models; authors of both studies [24, 55] provided accuracy data for M30 at seven threshold values we selected based on the data from the present meta-analysis (133, 200, 248, 292, 356, 395, and 464 U/L). This allowed us to apply the multiple thresholds model (15 thresholds), to calculate an AUC of 0.73 (95% CI: 0.57–0.85), mean sensitivity of 0.63 (95% CI: 0.39–0.82) and mean specificity of 0.73 (95% CI: 0.51–0.88) at a Youden-threshold value of 371 U/L.

### Fibrosis

#### Accuracy of CK-18 M30 in detecting significant and advanced fibrosis

We identified several studies that investigated CK-18 for fibrosis staging. For significant fibrosis, we included a single threshold value (ranging from 122 to 285 U/L) from five studies [27, 43, 47, 55, 59] with a total of 1,155 participants (554 had significant fibrosis) (S6 Fig in S1 File). The resulting AUC was 0.68. See S7A Fig in S1 File for SROC curve and corresponding 95% CI and prediction region. One study [50] assessed the ability of M65 to detect significant fibrosis; at a threshold of 244 U/L, sensitivity was 0.71 for a specificity of 0.71 (AUC: 0.74).

For advanced fibrosis, five studies [27, 42, 43, 51, 55] were included in the meta-analysis (1,513 participants, 313 with advanced fibrosis) (S8 Fig in S1 File). We calculated an AUC of 0.75, with included threshold values ranging from 216 to 396 U/L (see S7B Fig in S1 File). One study had to be excluded from the meta-analysis of both significant and advanced fibrosis due to discrepancies in the 2x2 contingency table [30].

### Multimarker models including CK-18

Thirteen studies additionally used CK-18 as an ingredient of a multimarker model (Table 4). There was greatest interest in detecting NASH (8/13 studies), with AUCs among the eight models ranging from 0.79 to 0.96. The highest performance was observed in NASH-score (BMI, alanine aminotransferase (ALT), aspartate aminotransferase (AST), alkaline phosphatase (ALP), HOMA-IR, M65 and adiponectin), which produced an AUC of 0.96 [57].

**Table 4:** Summary of studies that additionally included CK-18 in multimarker model.

Author	Target condition and population	Scoring system	Ingredients	AUC
1. **Anty 2010**	NAFLD grading among morbidly obese	The Nice Model	Metabolic syndrome, ALT, CK-18	Training: 0.88 Validation: 0.83
2. **Boursier 2018**	Fibrotic NASH among NAFLD	MACK-3	HOMA, AST, CK-18	Validation: 0.85
3. **Cao 2013**	NASH among NAFLD		ALT, platelets, M30, and TG	0.92
4. **Chuah 2019**	Fibrotic NASH among NAFLD	MACK-3	HOMA, AST, CK-18	0.80
5. **Ergelen 2015**	Fibrosis among NAFLD		TE, M30	F ≥2: 0.89 F ≥3: 0.93
6. **Grigorescu 2012**	NASH among NAFLD		M65, IL-6 and adiponectin	0.90
7. **Pirvulescu 2012**	NASH (including borderline NASH) among morbidly obese patients	NASH-score	BMI, ALT, AST, ALP, HOMA-IR, M65, and adiponectin	0.96
8. **Rosso 2016**	Fibrosis among NAFLD		TE, M30	F ≥2: 0.84 F ≥3: 0.87
9. **Tada 2018**	NASH among NAFLD	FIC-22	FIB-4 and CK-18	0.82
10. **Tamimi 2011**	NASH among NAFLD		Soluble fas and CK-18	Training: 0.93 Validation 0.79
11. **Yang 2015**	NASH among NAFLD		M30†, FGF-21, IL-1Ra, PEDF, and OPG	Training NPV: 0.76 and PPV: 0.85 Validation NPV: 0.80 and PPV: 0.76
12. **Younossi 2008**	NASH among NAFLD		M30 and M65, adiponectin, resistin	Training: 0.91 Validation: 0.73
13. **Younossi 2011**	NASH among NAFLD	NAFLD diagnostic panel	Diabetes, gender, BMI, triglycerides, M30, and M65	NASH: 0.81

NAFLD: non-alcoholic fatty liver disease, NASH: non-alcoholic steatohepatitis, ALT: alanine aminotransferase, CK-18: cytokeratin 18, AST: aspartate aminotransferase, TG: trigylceride, HOMA-IR: homeostatic model assessment for insulin resistance, TE: transient elastography, IL-6: interleukin 6, BMI: body max index, FGF-21: fibroblast growth factor 21, IL-1Ra: interleukin-1 receptor antagonist, NPV: negative predictive value, PPV: positive predictive value, PEDF: pigment epithelium-derived factor, OPG: osteoprotegerin.

† Unit of measure for M30 is ng/L.

One model was developed with the aim of detecting fibrotic NASH [55]. Composed of three ingredients (HOMA, AST and CK-18) the AUC from the validation group (n = 846) was 0.85. MACK-3 had an AUC of 0.80 when evaluated in a separate study [24].

Two studies [27, 43] investigated the combined use of M30 with transient elastography (TE) (FibroScan) to detect fibrosis. One study found combining TE and M30 to detect significant (AUC: 0.89) and advanced fibrosis (AUC: 0.93) did not significantly improve the diagnostic ability from either TE or CK-18 as a stand-alone test [27]. Another study, however, found some improvement in AUC by combining M30 to TE compared to TE alone; in adding M30 they found an improvement in AUC by 0.03 for significant fibrosis, and 0.05 for advanced fibrosis [43].

### Sensitivity analysis

A sensitivity analysis was conducted excluding four studies with two or more domains of high risk of bias or applicability concerns [9, 10, 31, 40] for M30 and NASH. The AUC was 0.75 (95% CI: 0.68–0.81), with a mean sensitivity of 0.62 (95% CI: 0.51–0.72), and mean specificity of 0.78 (95% CI: 0.66–0.86).

We identified four studies that used an ELISA assay that was not from PEVIVA [40, 42, 52, 53]. Among the 18 studies that used the M30 Apoptosense ELISA by PEVIVA, the AUC was 0.74 (95% CI: 0.67; 0.80), with paired sensitivity and specificity of 0.60 (95% CI: 0.49; 0.70) and 0.80 (95% CI: 0.70; 0.87), respectively. We additionally conducted sensitivity analysis solely among studies that included biopsy-proven NAFLD patients (19/22 studies for M30 and NASH), and found an AUC of 0.74 (95% CI: 0.67; 0.80). No marked differences were observed when excluding studies with high risk of bias or applicability concerns, different ELISA assays or cohorts with clinical suspicion of NAFLD.

## Discussion

### Main findings

Among NAFLD adults, the diagnostic accuracy of M30 to distinguish NASH from NAFL was under the minimally acceptable performance level, fixed a priori at AUC of 0.80. More promising results were observed for M65 and NASH, although it is of note that only six studies could be included in this meta-analysis, compared to 22 for M30. The superior performance of M65 should further be interpreted with caution, as its ability to detect fibrotic NASH, the most clinically relevant target condition, is limited.

At lower prevalence, mirroring primary care settings, high NPVs above 0.85 were achieved for both M30 and M65 antigens at fixed sensitivity and specificity values above 0.80 (Tables 2 and 3).

Our meta-analysis on the accuracy of M30 in detecting fibrotic NASH also showed modest performance. MACK-3 showed more promise for detecting fibrotic NASH, but the evidence is still limited to two studies, and the model presents with limitations such as adequate performance among subgroups with metabolic syndrome and a large gap of patients who lie between the high and low threshold values [24, 55].

Results for both significant and advanced fibrosis were below the minimally acceptable performance level, demonstrating sub-optimal ability of M30 to function as a stand-alone test for fibrosis staging, even more so when considering the available accurate elastography methods and multimarker models for detecting liver fibrosis.

As expected, we observed a wide range of reported threshold values for both CK-18 antigens. This can be explained by the variability of methods employed for choosing a threshold and general lack of established recommendations. With our meta-analysis we suggest high and low thresholds for M30 and M65, which can be selected in accordance to the intention of use (ruling-in or ruling-out NASH). It is of note that the threshold suggestions for the M30 and M65 antigens are strictly for results produced by the PEVIVA assays, as it is understood that different CK-18 assays show poor inter-test reliability and majority of our studies used CK-18 assays from PEVIVIA [42].

### Strengths and limitations

By employing novel meta-analytical methods, we were able to incorporate all data available in the primary studies, eliminating arbitrary selection of a single threshold for our meta-analyses. This allowed greater freedom to investigate which clinical setting would optimize the use of CK-18. A more comprehensive evaluation of the clinical performance, including projections of accuracy data (sensitivity, specificity, PPV, NPV) in various prevalence settings was possible. The multiple thresholds model further allowed us to assess the diagnostic accuracy of CK-18 at threshold values not investigated in the original studies. We were however limited in the sense that the data projected by our models are based on the cumulative distribution of CK-18 in the diseased and non-diseased populations of the primary studies, which had higher prevalence than one would expect in a primary care setting.

The approach for selecting either a single ‘optimal’ threshold value or a set of thresholds were very heterogeneous in our included studies. While some used the Youden or equivalent methods, others chose to optimize either the sensitivity or specificity, and a concerning few did not report how a threshold value was calculated. This was however anticipated as there is no recommended threshold for CK-18. We further observed sparse reporting of the histological procedure, including quality of biopsies and expertise of histological evaluation (S6 Table in S1 File), which raises concerns regarding the reliability of the reference standard test.

### In context of published literature

For M30 and NASH (22 studies), we found lower diagnostic accuracy compared to previous meta-analyses. He (2017) (14 studies) reported an AUC of 0.82 [60]; Kwok (2014) (seven studies) reported a summary sensitivity of 0.66, at a specificity of 0.82 [13]; Chen [15] (nine studies) found an AUC of 0.84 [15]; and Musso (2010) (nine studies) found an AUC of 0.82 [61]. Parameters such as mean age, BMI and disease prevalence were not sources of major heterogeneity between the present and previously published meta-analyses [61]. Our meta-analysis did however include a greater number of studies, incorporating more recent publications with lower performance. Among the six studies published after 2017, the AUC ranged between 0.59 and 0.77 for M30 in detecting NASH, a noticeable drop compared to pioneering work from 2008–10 (AUC: 0.71 to 0.88). The lowest AUC (0.59) was found in the largest study (N = 846) conducted in 2018. Interestingly, this study also found M30 to be most accurate in detecting patients with fibrotic NASH, achieving an AUC of 0.72 [55]. In parallel with the incrementally less impressive results, the excitement for CK-18 as a NAFLD biomarker has tempered with each subsequent study, serving as an exemplar of the entire biomarker space.

The only other meta-analysis performed on the diagnostic ability of both M30 and M65 concluded that both antigens had similar ability to distinguish NASH from NAFL (M30 had AUC of 0.82, M65 had AUC of 0.80) [60]. Among the three studies that investigated both M30 and M65 within the same cohort, all found better performance for M65 compared to M30 [9, 26, 57]. Although M30 has been more popularly studied as a diagnostic biomarker for NASH, our meta-analysis demonstrates the need for more evidence to establish the performance of M65. Further studies conducting head-to-head comparisons of M30 and M65 within the same cohort would be valuable for assessing superior performance of either antigen.

Fibrotic NASH has become an emerging target condition of interest in NAFLD research [17]. Despite the established role of hepatocyte apoptosis in the progression of liver damage [11], there have been contradictory opinions regarding the usefulness of CK-18 for fibrosis staging. Our results showed limited ability of CK-18 to function as a stand-alone test for detecting fibrotic patients compared to existing biomarkers.

Even still, the involvement of CK-18 in the disease pathway of NAFLD indicates potential for CK-18 to be used in combination with other biomarkers. Several promising models that included CK-18 (M30 and/or M65) were identified in our systematic review, most of which exceeded the minimally acceptable performance level of an AUC ≥0.80. Unfortunately, most models are limited to a single validation within the original studies with the exception of M30 with TE, and MACK-3, which raises the concern of how well the models would perform in practice. Additional validation studies for the proposed multimarker models should be conducted to ensure reliability of their performance. We do acknowledge that other studies including CK-18 in a composite scoring system may exist, despite not being eligible for inclusion in the present systematic review [62, 63]. For example, a recent study developed a model for distinguishing NASH from NAFL, finding an AUC of 0.73 (0.66–0.81), with even better accuracy for detecting advanced fibrosis [63].

### Implications for current practice and future research

Both the EASL-EASD-EASO and Asia-Pacific Working Party guidelines suggest that CK-18 has limited ability to function as a stand-alone test for distinguishing NASH from NAFL given its modest performance [12, 14]. However, in a setting with 20% prevalence, a sensitivity of 0.90 and a NPV of 0.91 were achieved at a threshold value of 127 U/L (M30), demonstrating high negative values for ruling-out those without NASH. In such a scenario CK-18 could be of value as a first-line test at a primary care level for further evaluation by a specialist, even more so when considering the low cost and accessibility. This however comes at the cost of lower specificity, resulting in a high number of false positive results, as well as the compromise of 62% misclassified patients in the same setting with 20% prevalence. Alternatively, should CK-18 be used to rule-in NASH, a higher threshold of 399 U/L would be more appropriate. The trade-off between sensitivity and specificity as well as predictive values should be considered before selecting a threshold to be use in clinical practice, as a substantial number of patients without NASH could be referred for further, more invasive and risky evaluation.

CK-18 can potentially improve risk stratification in combination with other synergistic markers, such as TE or NFS, by testing for elevated M30 levels among patients under the low threshold or in patients with intermediate TE/NFS values (between the high and low threshold) [64]. In the study by Liebig et al., risk stratification was considerably improved with this approach, showing more than 70% of patients with low TE/NFS but elevated M30 revealing presence of NASH (mostly with fibrosis). As with CK-18, other highly validated tests also run the risk of misclassified patients, for example, those with low or intermediate risk by TE who would not be considered for a biopsy despite presence of NASH. In such a step-wise diagnostic regime, a high cut-off for M30 should be selected to optimize specificity and rule-in those with NASH.

## Supporting information

S1 File (DOCX)
