# Supplementary material for: Accuracy of cytokeratin 18 (M30 and M65) in detecting non-alcoholic steatohepatitis and fibrosis: A systematic review and meta-analysis
Source: PLoS One. 2020 Sep 11;15(9):e0238717. doi: 10.1371/journal.pone.0238717 (PMC7485872; doi:10.1371/journal.pone.0238717)
Supplement: S1 File — (DOCX) [file pone.0238717.s001.docx]

# **Supporting information**

## Tables

### S1 Table. PRISMA 2009 Checklist

| **Section/topic** | **#** | **Checklist item** | **Reported on page #** |
| --- | --- | --- | --- |
| **TITLE** | | |  |
| Title | 1 | Identify the report as a systematic review, meta-analysis, or both. | 1 |
| **ABSTRACT** | | |  |
| Structured summary | 2 | Provide a structured summary including, as applicable: background; objectives; data sources; study eligibility criteria, participants, and interventions; study appraisal and synthesis methods; results; limitations; conclusions and implications of key findings; systematic review registration number. | 3 |
| **INTRODUCTION** | | |  |
| Rationale | 3 | Describe the rationale for the review in the context of what is already known. | 5-6 |
| Objectives | 4 | Provide an explicit statement of questions being addressed with reference to participants, interventions, comparisons, outcomes, and study design (PICOS). | 6 |
| **METHODS** | | |  |
| Protocol and registration | 5 | Indicate if a review protocol exists, if and where it can be accessed (e.g., Web address), and, if available, provide registration information including registration number. | 6 |
| Eligibility criteria | 6 | Specify study characteristics (e.g., PICOS, length of follow-up) and report characteristics (e.g., years considered, language, publication status) used as criteria for eligibility, giving rationale. | 7-8 |
| Information sources | 7 | Describe all information sources (e.g., databases with dates of coverage, contact with study authors to identify additional studies) in the search and date last searched. | 7-8 |
| Search | 8 | Present full electronic search strategy for at least one database, including any limits used, such that it could be repeated. | 7 |
| Study selection | 9 | State the process for selecting studies (i.e., screening, eligibility, included in systematic review, and, if applicable, included in the meta-analysis). | 7 |
| Data collection process | 10 | Describe method of data extraction from reports (e.g., piloted forms, independently, in duplicate) and any processes for obtaining and confirming data from investigators. | 8 |
| Data items | 11 | List and define all variables for which data were sought (e.g., PICOS, funding sources) and any assumptions and simplifications made. | 7 |
| Risk of bias in individual studies | 12 | Describe methods used for assessing risk of bias of individual studies (including specification of whether this was done at the study or outcome level), and how this information is to be used in any data synthesis. | 8 |
| Summary measures | 13 | State the principal summary measures (e.g., risk ratio, difference in means). | 9-10 |
| Synthesis of results | 14 | Describe the methods of handling data and combining results of studies, if done, including measures of consistency (e.g., I^2^) for each meta-analysis. | 9-10 |

| Risk of bias across studies | 15 | Specify any assessment of risk of bias that may affect the cumulative evidence (e.g., publication bias, selective reporting within studies). | 10 |
| --- | --- | --- | --- |
| Additional analyses | 16 | Describe methods of additional analyses (e.g., sensitivity or subgroup analyses, meta-regression), if done, indicating which were pre-specified. | 9-10 |
| **RESULTS** | | |  |
| Study selection | 17 | Give numbers of studies screened, assessed for eligibility, and included in the review, with reasons for exclusions at each stage, ideally with a flow diagram. | 10 |
| Study characteristics | 18 | For each study, present characteristics for which data were extracted (e.g., study size, PICOS, follow-up period) and provide the citations. | 11-13 |
| Risk of bias within studies | 19 | Present data on risk of bias of each study and, if available, any outcome level assessment (see item 12). | 14 |
| Results of individual studies | 20 | For all outcomes considered (benefits or harms), present, for each study: (a) simple summary data for each intervention group (b) effect estimates and confidence intervals, ideally with a forest plot. | 14-20 |
| Synthesis of results | 21 | Present results of each meta-analysis done, including confidence intervals and measures of consistency. | 14-20 |
| Risk of bias across studies | 22 | Present results of any assessment of risk of bias across studies (see Item 15). | 20 |
| Additional analysis | 23 | Give results of additional analyses, if done (e.g., sensitivity or subgroup analyses, meta-regression [see Item 16]). | 20 |
| **DISCUSSION** | | |  |
| Summary of evidence | 24 | Summarize the main findings including the strength of evidence for each main outcome; consider their relevance to key groups (e.g., healthcare providers, users, and policy makers). | 20-21 |
| Limitations | 25 | Discuss limitations at study and outcome level (e.g., risk of bias), and at review-level (e.g., incomplete retrieval of identified research, reporting bias). | 21-22 |
| Conclusions | 26 | Provide a general interpretation of the results in the context of other evidence, and implications for future research. | 22-24 |
| **FUNDING** | | |  |
| Funding | 27 | Describe sources of funding for the systematic review and other support (e.g., supply of data); role of funders for the systematic review. | 24-25 |

### S2 Table. MEDLINE search strategy

| # | Search strategy | Results |
| --- | --- | --- |
| 1 | exp Fatty Liver/ | 27851 |
| 2 | (NAFL* or NASH*).mp. | 16307 |
| 3 | "non-alcoholic fatty liver disease*".mp. | 11950 |
| 4 | (((fatty or fat or steato*) adj3 (liver* or hepat*)) or steatohepat* or (visceral adj2 steato*)).ti,ab. | 42353 |
| 5 | 1 or 2 or 3 or 4 | 53450 |
| 6 | exp "sensitivity and specificity"/ or exp "mass screening"/ or "reference values"/ or "false positive reactions"/ or "false negative reactions"/ or specificit$.tw. or screening.tw. or false positive$.tw. or false negative$.tw. or accuracy.tw. or predictive value$.tw. or reference value$.tw. or roc$.tw. or likelihood ratio$.tw. or predictive value$.tw. | 1782749 |
| 7 | exp BIOMARKERS/ | 658965 |
| 8 | (biomarker$ or marker$).ti,ab,kf,rn. | 990839 |
| 9 | (test* or measure* or level* or diagnos* or ratio or score*).ti,ab. | 9540131 |
| 10 | ((biomarker* or marker*) adj4 (test* or measure* or level* or ratio or score*)).ti,ab. | 61523 |
| 11 | Laboratory Test$.ti,ab,kf. | 37954 |
| 12 | (Cytokeratin-18 or Keratin-18 or CYK18 or CYK-18 or KRT18 or KRT-18).ti,ab,kf. | 1865 |
| 13 | exp cytokeratin 18/ | 862 |
| 14 | ((Spectroscopy or LS23 or spectrometer) and DiaFir).ti,ab,kw. or MIR-FEWS.ti,ab,kf. | 1 |
| 15 | ("enhanced liver fibrosis" or ELFscore or ELFtest).ti,ab,kf. | 127 |
| 16 | (Glycomics-based or Glyco-Liver or N-glycans or Nglycans or (Glyco* adj3 (profile or test or measure))).ti,ab,kf. | 5736 |
| 17 | ((SOMAscan or SOMAmers) adj4 (V4 or plex)).mp. | 1 |
| 18 | (miR-122 or miR-34a or (miR122 or miR34a) or (micro RNA 122 or micro RNA 34a)).ti,ab,kf. | 2674 |
| 19 | (Alpha-2 Macroglobulin or A2M or Alpha2 Macroglobulin or Alpha2-Macroglobulin or A2 Macroglobulin or A2-macroglobulin or a2-macroglobulin or a2 macroglobulin or a2macroglobulin).ti,ab,kf. | 6318 |
| 20 | (haptoglobin or HP or Hpt or a2-glycoprotein).ti,ab,kf. | 28469 |
| 21 | (apolipoprotein a1 or APOA1 or Apolipoprotein A-I or apoA-I or apo A-I or apoA-1 or apo A-1 or apo-AI or Apo-A1).ti,ab,kf. | 13088 |
| 22 | (((OWLiver or OWL) adj2 test*) or (OWL adj2 metabolomic*)).ti,ab,kf. | 12 |
| 23 | (type III pro-collagen or type III procollagen or type 3 pro-collagen or type 3 procollagen or Pro-C3 or Proc3).ti,ab,kf. | 1238 |
| 24 | ((7S domain adj3 collagen type IV) or P4NP_7S or P4NP7S or P4NP-7S).ti,ab,kf. | 20 |
| 25 | ((((A2 or A9) adj3 (fibrogenesis or fibrolysis)) or ((fibrogenesis or fibrolysis) adj3 marker*)) and (A2 or A9)).ti,ab,kf. | 1 |
| 26 | (((extracellular matrix or matricellular or ECM) adj2 molecules) and (A2 or A9)).ti,ab,kf. | 4 |
| 27 | (type VI pro-collagen or type VI procollagen or type 6 pro-collagen or type 6 procollagen or Pro-C6 or Pro C6 or Proc6).ti,ab,kf. | 17 |
| 28 | ((nafld or bard or ferritin* or fibrosis) adj4 (test* or measure* or level* or ratio or score*)).ti,ab,kf. | 17326 |
| 29 | FIB-4.ti,ab. | 689 |
| 30 | ((glutamic-pyruvic transaminase or glutamic-oxaloacetic transaminase or sgot or sgpt or alt or ast) adj4 (test* or measure* or level* or ratio or score*)).ti,ab. | 14382 |
| 31 | (alanine adj2 (aminotransferase or transaminase) adj4 (test* or measure* or level* or ratio or score*)).ti,ab. | 9188 |
| 32 | ((ast-to-platelet ratio index or apri or elf or enhanced liver fibrosis or nash) adj4 (panel or test* or measure* or level* or score*)).ti,ab. | 1237 |
| 33 | ((Aspartate or AST or Aminotransferase) adj3 Platelet adj2 ratio adj2 index).ti,ab. | 737 |
| 34 | (APRI or APR-index or APRindex or ("AST/platelet" adj3 "ratio index")).ti,ab. | 997 |
| 35 | (fibro-test* or fibrometer or fibro-meter* or fib4 or fib-4).ti,ab,kf. | 878 |
| 36 | Hepascore.mp. | 66 |
| 37 | (fibroblast activation protein* or FAP).mp. | 4961 |
| 38 | ((Apolipoprotein adj3 F) or Apo-F or ApoF or Apo F).mp. | 64 |
| 39 | 7 or 8 or 9 or 10 or 11 or 12 or 13 or 14 or 15 or 16 or 19 or 20 or 21 or 22 or 23 or 24 or 25 or 26 or 27 or 28 or 29 or 30 or 31 or 32 or 33 or 34 or 35 or 36 or 37 or 38 | 10060746 |
| 40 | 5 and 6 and 39 | 3544 |
| 41 | exp animals/ not humans/ | 4486179 |
| 42 | 40 not 41 | 3238 |

### S3 Table. Histological scoring systems developed to characterize changes in NAFLD progression

| Brunt criteria (Necroinflammatory Grading System for Steatohepatitis) | A system for semi-quantitative evaluation for the unique lesions recognized for NASH, developed for NASH and does not encompass the entire spectrum of NAFLD. | |
| --- | --- | --- |
|  | Mild, grade 1 | Steatosis (predominantly macrovesicular) involving up to 66% of biopsy; may see occasional ballooned zone 3 hepatocytes; scattered rate intra-acinar pmn’s 6 intraacinar lymphocytes; no or mild portal chronic inflammation. |
|  | Moderate, grade 2 | Steatosis of any degree; ballooning of hepatocytes (predominantly zone 3) obvious; intra-acinar pmn’s noted, may be associated with zone 3 pericellular fibrosis; portal and intra-acinar chronic inflammation noted, mild to moderate. |
|  | Severe, grade 3 | Panacinar steatosis; ballooning and disarray obvious, predominantly in zone 3; intra-acinar inflammation noted as scattered pmn’s, pms’s associated with ballooned hepatocytes 6 mild chronic inflammation; portal chronic inflammation mild or moderate, not marked. |
| Matteoni criteria (The original criteria for NAFLD subtypes) | Developed to encompass the entire spectrum of NAFLD. NAFLD types 3 and 4 were considered to be NASH | |
|  | NAFLD type 1 | Steatosis alone |
|  | NAFLD type 2 | Steatosis with lobular inflammation only |
|  | NAFLD type 3 | Steatosis with hepatocellular ballooning |
|  | NAFLD type 4 | Steatosis with Mallory-Denk bodies or fibrosis |
| NAFLD Activity Score (NAS) | A semi-quantitative scoring system, on a scale of 0 to 8, for assessing the range of histological features of NAFLD. Comprised of 14 histological features: steatosis (0-3), lobular inflammation (0-3), hepatocellular ballooning (0-2). | |
|  | Steatosis | Score 0: <5% (minimal)  Score 1: 5-33% (mild)  Score 2: 34-66% (moderate)  Score 3: >66% (severe) |
|  | Lobular inflammation | Score 0: none  Score 1: <2 foci / 20× field  Score 2: 2-4 foci / 20× field  Score 3: >4 foci / 20× field |
|  | Hepatocellular ballooning | Score 0: none  Score 1: few (mild)  Score 2: many (moderate-marked) |
|  | Fibrosis | Score 0: none  Score 1: perisinusoidal or periportal (1a: mild, zone 3, perisinusoidal, 1b: moderate, zone 3, perisinusoidal, 1c: portal/periportal)  Score 2: perisinusoidal and portal/periportal  Score 3: bridging fibrosis  Score 4: cirrhosis |
| SAF score (steatosis,  activity, fibrosis) | SAF score dissociates grade of steatosis, grade of activity, and stage of fibrosis | |
|  | The steatosis score (S) | From 0 to 3 (S0: <5%; S1: 5%-33%, mild; S2: 34%-66%, moderate; S3: >67%, marked). |
|  | Activity grade (A) | From 0-4, is the unweighted addition of hepatocyte ballooning (0-2)  and lobular inflammation (0-2)  A0 (A ¼ 0) no activity, A1 (A ¼ 1), mild activity, A2 (A ¼ 2), moderate activity, A3 severe activity. |
|  | Stage of fibrosis (F) | Stage 0 (F0) (none); stage 1 (F1): 1a or 1b perisinusoidal zone 3 or  1c portal fibrosis, stage 2 (F2): perisinusoidal and periportal  fibrosis without bridging, stage 3 (F3): bridging fibrosis and stage 4 (F4): cirrhosis |
| Younossi criteria | 1 | Any degree of steatosis along with centrilobular ballooning and/or Mallory-Denk bodies |
|  | 2 | Any degree of steatosis along with centrilobular pericellular/perisinusoidal fibrosis or bridging fibrosis in the absence of another identifiable cause |

### S4 Table. Histological scoring systems for liver fibrosis

| Stages | F0 | F1 | F2 | F3 | F4 | F5 | F6 |
| --- | --- | --- | --- | --- | --- | --- | --- |
| 7-point scoring systems | | | | | | | |
| EPoS Staging System | No fibrosis | Portal or perisinusoidal fibrosis | Central or portal fibrosis + lobular fibrosis or portal + central fibrosis | Few septa (no more than 2 /10mm length of biopsy) | Many septa (> 2) without nodule | Many septa with few nodules | Annular fibrosis with complete nodulation |
| Ishak Score | No fibrosis | Some portal tract fibrotic ± short fibrous septa | Most portal tract fibrotic ± short fibrous septa | Portal tract fibrotic with occasional portal to portal bridging | Portal tract fibrotic with marked portal to portal and portal to central bridging | Marked portal to portal and/or portal to central with occasional nodules | Cirrhosis |
| 5-point scoring systems | | | | | | | |
| NASH CRN Fibrosis Stage | No fibrosis | Portal or perisinusoidal  1A: Mild perisinusoidal fibrosis (zone 3)  1B: Moderate perisinusoidal fibrosis (zone 3)  1C: Portal/periportal fibrosis | Perisinusoidal and portal/periportal fibrosis | Bridging | Cirrhosis | - | - |
| METAVIR Fibrosis Score | No fibrosis | Portal fibrosis without septa | Septal fibrosis (portal-portal) | Septal fibrosis (portal-central) | Cirrhosis | - | - |
| Knodell Fibrosis Stage | No fibrosis | Fibrous portal expansion |  | Bridging fiborisis (portal-portal or portal-central linkage) | Bridging fiborisis (portal-portal or portal-central linkage) | - | - |
| Scheuer System | No fibrosis | Enlarged fibrotic portal tracts | Periportal or portal-portal septa but intact architecture | Fibrosis with architectural distortion but no obvious cirrhosis | Probable or definite cirrhosis | - | - |
| Batts-Ludwig System | No fibrosis | Fibrous portal expansion | Periportal or rare portal-portal septa | Fibrous septa with architectural distortion; no obvious cirrhosis | Cirrhosis | - | - |
| International Association for Study of the Liver (IASL)  scoring system | No fibrosis | Mild fibrosis (Fibrous portal expansion) | Moderate fibrosis (Few bridges or septa) | Severe fibrosis (Numerous bridges or septa) | Cirrhosis | - | - |

Not all scoring systems are equivalent to each other.

### S5 Tables. Conversion grd for liver fibrosis staging

| Fibrosis  Distribution | NASH CRN fibrosis stage | Ishak  Fibrosis stage | METAVIR fibrosis stage | Knodell Fibrosis stage | Scheuer Fibrosis stage | International Association for Study of the Liver (IASL)  scoring system | The Batts-Ludwig system | EPoS staging system |
| --- | --- | --- | --- | --- | --- | --- | --- | --- |
| No excess fibrosis | F0 | F0 | F0 | F0 | F0 | F0 | F0 | F0 |
| Portal or perisinusoidal | F1 | F1 | F1 | F1 | F1 | F1 | F1 | F1 |
| Portal and perisinusoidal | F2 | F2 | F1 | F1 | F1 | F1 | F2 | F2 |
| Bridging | F3 | F3-4 | F2-3 | F3 | F2-3 | F2-3 | F3 | F3-4 |
| Nodules | F4 | F5- 6 | F4 | F4 | F4 | F4 | F4 | F5- 6 |

### S6 Table. Summary of index test and liver biopsy characteristics

| Study ID | CK-18 (M30/ M65) | Index test kit | Target condition (definition) | Scoring system | Biopsy characteristics | | | Biopsy reviewers | Blinded to clinical data |
| --- | --- | --- | --- | --- | --- | --- | --- | --- | --- |
|  |  |  |  |  | Needle gauge (mm) | Length (mm) | No. of portal tracks |  |  |
| 1. Aida 2014 | M30 | Peviva | NASH (NAS ≥5) | NAFLD Activity Score | 16 | 18 | 10 | Single experienced pathologist | Yes |
| 1. Anty 2010 | M30 | Peviva | NASH (NAS ≥5) | NAFLD Activity Score | - | - | - | Two hepatopathologists | Yes |
| 1. Boursier 2018 | M30 | Peviva | NASH (NAS ≥5)  Fibrotic NASH (NAS ≥4, F ≥ 2)  Sig. fibrosis (F ≥2)  Adv. fibrosis (F ≥3) | NAFLD Activity Score  NASH CRN | 14, 16 | - | - | Senior expert hepatopathologist | Yes |
| 1. Cao 2013 | M30 | Peviva | NASH (NAS ≥5) | NAFLD Activity Score | - | 20 | 13 | Two pathologists | Yes |
| 1. Chan 2014 | M30 | Peviva | NASH (NAS ≥5) | NAFLD Activity Score | 18 | 15 | 8 | Single experienced histopathologist | Yes |
| 1. Chuah 2019 | M30 | Peviva | Fibrotic NASH (NAS ≥4 and F ≥2) | NAFLD Activity Score | 18 | 15 | 8 | Single experienced histopathologist | Yes |
| 1. Cusi 2013 | M30 | Peviva | NASH (NAS ≥5) | NAFLD Activity Score | - | - | - | Single experienced pathologist | Yes |
| 1. Diab 2008 | M30 | Peviva | NASH (NAS ≥5) | NAFLD Activity Score | - | - | - | Single experienced hepatopathologist | Yes |
| 1. Dvorak 2014 | M30/M65 | Peviva | NASH (NAS ≥5) | NAFLD Activity Score | - | - | - | Single pathologist | Yes |
| 1. Ergelen 2015 | M30 | Peviva | Sig. fibrosis (F ≥2)  Adv. fibrosis (F ≥3) | NASH CRN | - | 20 | 11 | - | Yes |
| 1. Feldstein 2009 | M30 | Peviva | NASH (NAS ≥5) | NAFLD Activity Score | - | - | 17 | Study pathologists according to expertise | - |
| 1. Grigorescu 2012 | M65 | Peviva | NASH (NAS ≥5) | NAFLD Activity Score | - | - | - | Single senior hepatopathologist | Yes |
| 1. Hasegawa 2015 | M65 | Peviva | NASH (NAS ≥5) | NAFLD Activity Score | - | - | - | Two expert hepatopathologists | - |
| 1. Huang 2017 | M30 | Peviva | Sig. fibrosis (F ≥2)  Adv. fibrosis (F ≥3) | METAVIR | - | 20 | - | Single experienced hepatopathologist | Yes |
| 1. Joka 2011 | M30/M65 | Peviva | NASH (NAS ≥5) | NAFLD Activity Score | - | 24 | - | Single pathologist | - |
| 1. Kamada 2013 | M30 | Peviva | NASH (NAS ≥5) | NAFLD Activity Score | - | 15 | 6 | Two hepatopathologists | Yes |
| 1. Kawanka 2015 | M30 | Pivka | NASH (NAS ≥5) | NAFLD Activity Score | - | - | - | Two expert pathologists | - |
| 1. Kazankov 2016 | M30 | Peviva | NASH (NAS ≥5) | NAFLD Activity Score | - | - | 11 | Two experienced pathologists | Yes |
| 1. Kim 2013 | M30 | Peviva | NASH (Type 3-4) | Matteoni criteria | 16 | - | - | Two experienced hepatopathologists | Yes |
| 1. Kobayashi 2017 | M30 | Peviva | NASH (Type 3-4)  NASH with mild fibrosis (Type 3-4, F0-2) | Matteoni criteria | 17 | - | - | One hepatologist and one pathologist | Yes |
| 1. Liu 2016 | M30 | Peviva | NASH | SAF score | - | - | - | Single experienced hepatopathologist | Yes |
| 1. Malik 2009 | M30 | Diapharma Group | NASH (NAS ≥4)  Adv. fibrosis (F ≥3) | NAFLD Activity Score  NASH CRN | - | 20 | - | - | Yes |
| 1. Musso 2010 | M30 | - | NASH | - | - | - | - | - | - |
| 1. Papatheodoridis 2010 | M30 | Peviva | NASH (NAS ≥5) | NAFLD Activity Score | - | 26 | - | Single liver histopathologist | Yes |
| 1. Pimentel 2016 | M30 | Diapharma Group | NASH (NAS ≥5)  Adv. fibrosis (F ≥3) | NAFLD Activity Score  NASH CRN | - | 12 | - | Single experienced liver pathologist | Yes |
| 1. Pirvulescu 2012 | M30/M65 | Peviva | NASH & borderline NASH (NAS ≥3) | NAFLD Activity Score | - | 20 | 8 | Two hepatopathologists | - |
| 1. Rosso 2016 | M30 | Peviva | Sig. fibrosis (F ≥2)  Adv. fibrosis (F ≥3) | METAVIR | - | - | - | Single expert pathologist | - |
| 1. Shen 2012 | M30/M65 | Peviva | NASH (NAS ≥5) | NAFLD Activity Score | 16 | 19 | - | Two experienced pathologists | Yes |
| 1. Tada 2018 | M30 | Institute of Immunology, Tokyo, Japan | NASH (Type 3-4) | Matteoni criteria | 17 | - | - | Single pathologist specializing in NAFLD | Yes |
| 1. Tamimi 2011 | M30 | Peviva | NASH (NAS ≥5) | NAFLD Activity Score | - | - | - | Single experienced hepatopathologist | Yes |
| 1. Valva 2018 | M30 | Peviva | Sig. fibrosis (F ≥2) | NASH CRN | - | - | - | Two pathologists | Yes |
| 1. Wieckowska 2006 | M30 | Peviva | NASH (NAS ≥5) | NAFLD Activity Score | - | - | - | Single experienced hepatopathologist | Yes |
| 1. Yang 2015 | M30 | - | NASH (NAS ≥5) | NAFLD Activity Score | 16 | - | - | Two experienced pathologists | - |
| 1. Yilmaz 2007 | M30/M65 | Peviva | Sig. fibrosis (F ≥2) | NASH CRN | 16 | 25 | - | Single experienced pathologist | Yes |
| 1. Younes 2018 | M30 | Peviva | NASH (NAS ≥5)  Adv. fibrosis (F ≥3) | NAFLD Activity Score  NASH CRN | - | 25 | 11 | Single expert liver pathologist | Yes |
| 1. Younossi 2008 | M30 | AXXORA | NASH (NAS ≥5) | Younossi criteria | - | - | - | Single hepatopathologist | Yes |
| 1. Younossi 2011 | M30 | Peviva | NASH (steatosis, lobular inflammation, and ballooning degeneration with or without Mallory–Denk bodies and/or fibrosis) | - | - | - | - | Single experienced hepatopathologist |  |
| 1. Darweesh 2019 | M30 | Cusabio Biochemistry | Steatosis (S ≥2) | NAFLD Activity Score | - | 15 | >6 | Two experi- enced hepatic pathologists | Yes |
| 1. Liu 2019 | M30/M65 | Herui BioMed Company | NASH (NAS ≥5) | NAFLD Activity Score | - | - | - | Single liver pathologist | Yes |
| 1. Mohammed 2019 | M30 | Elabscience | NASH (NAS >5) | NAFLD Activity Score | 10-20 | 15 | - | Two well-trained histopathologists | Yes |
| 1. Zheng 2020 | M30 | Herui Biomed Company | NASH (NAS ≥4) | NAFLD Activity Score | - | - | - | Single liver pathologist | Yes |

Data not reported

## Figures


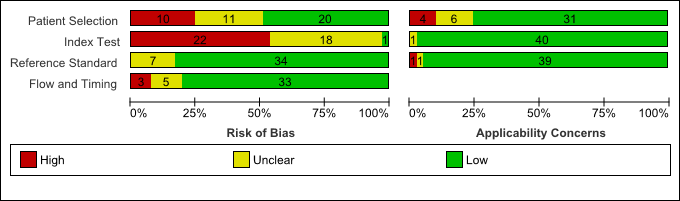


### S1 Fig. Graphical summary of the methodological quality of included studies using the QUADAS-2 tool


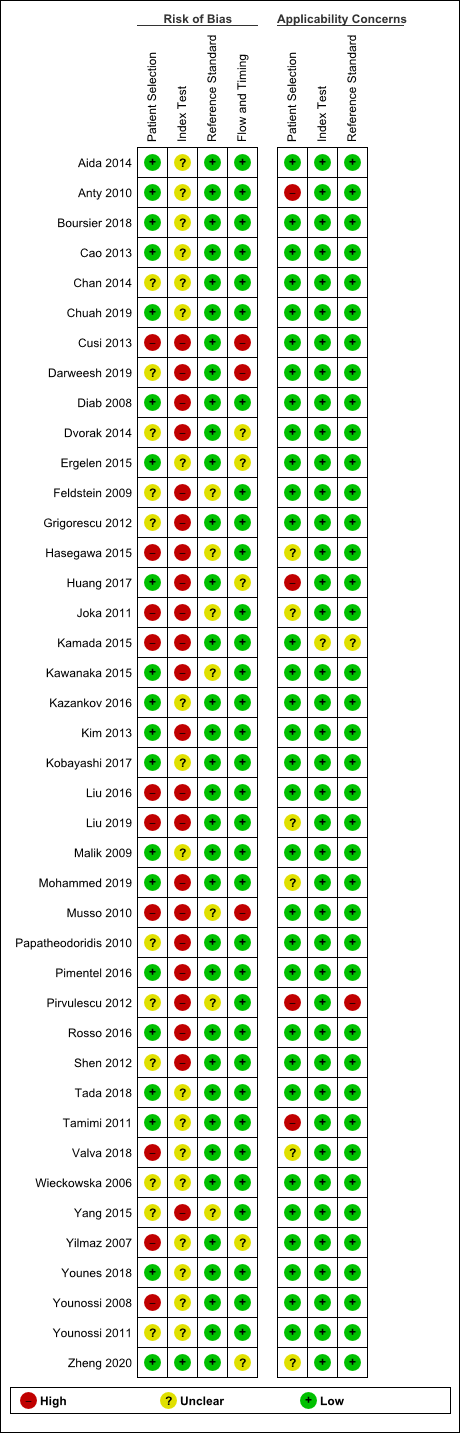


### S2 Fig. Methodological quality of each of the included studies per domain of the QUADAS-2 tool

### S3 FIg. Forest plot of studies investigating diagnostic accuracy of CK-18 (M30) in detecting NASH. TP: true positive, FP: false positive, FN: false negative, TN: true negative.


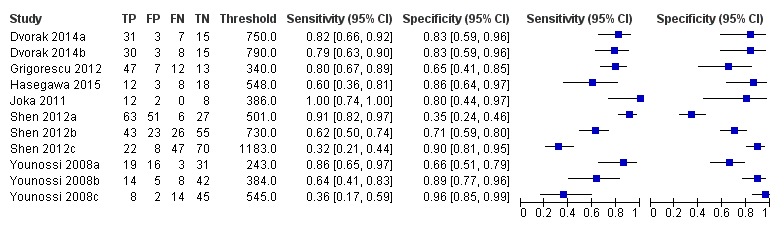


### S4 Fig. Forest plot of studies investigating diagnostic accuracy of CK-18 (M65) in detecting NASH. TP: true positive, FP: false positive, FN: false negative, TN: true negative.


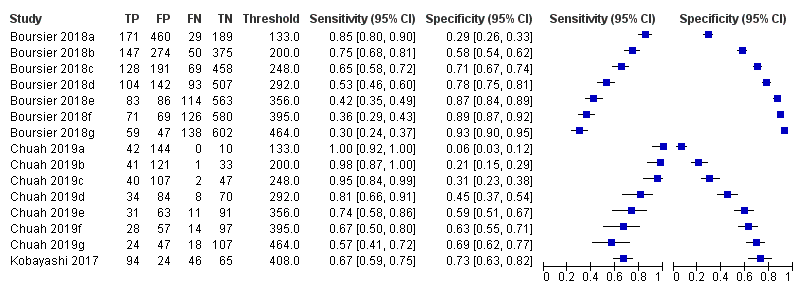


### S5 Fig. Forest plot of studies investigating diagnostic accuracy of CK-18 (M30) in detecting fibrotic NASH. TP: true positive, FP: false positive, FN: false negative, TN: true negative.


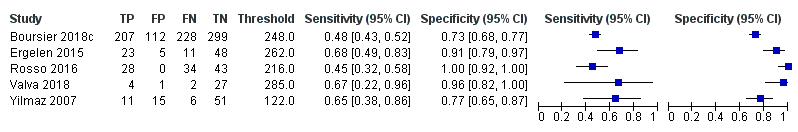


### S6 Fig. Forest plot of studies investigating diagnostic accuracy of CK-18 (M30) in detecting significant fibrosis. TP: true positive, FP: false positive, FN: false negative, TN: true negative.

1. B.


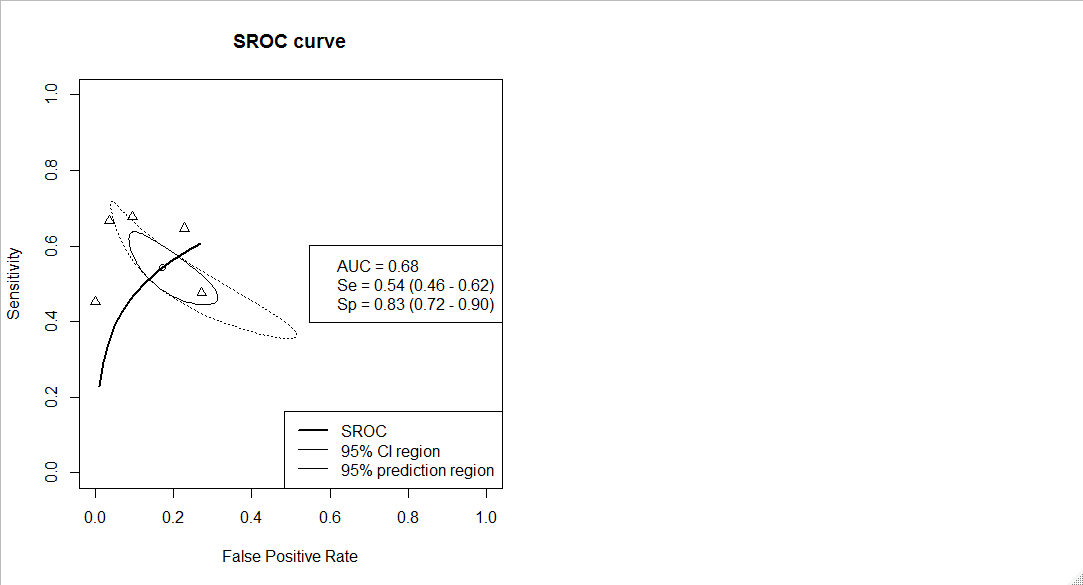

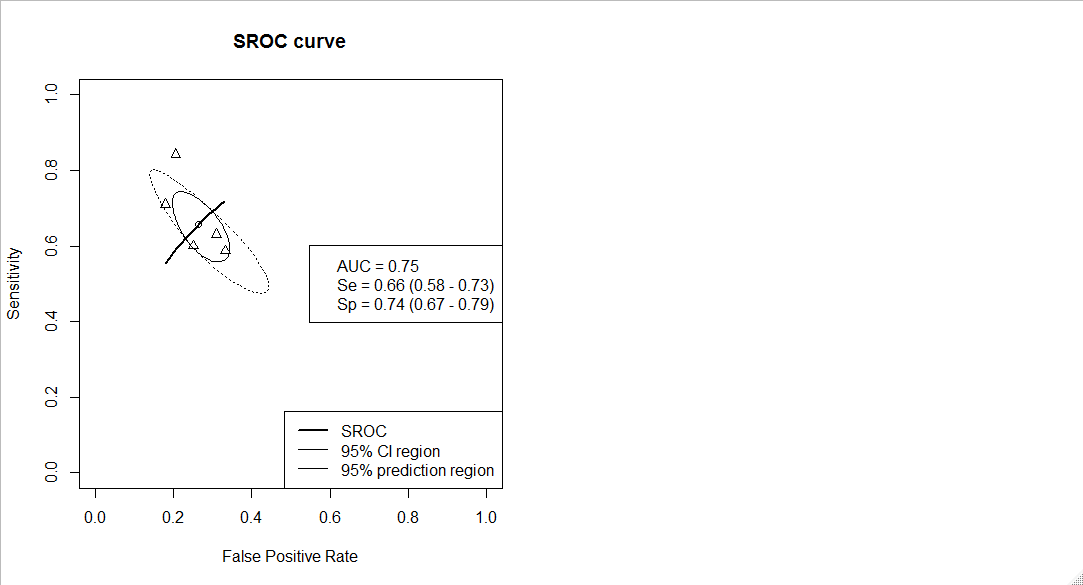


### S7 Fig. SROC curves for CK-18 M30 in detecting significant (A) and advanced (B) fibrosis. The x-axis indicates 1 – specificity, and the y-axis, sensitivity. The point in the SROC curve indicates the Youden-based threshold value. Each triangle represents a single threshold value reported from an included study. The solid eclipse represents the 95% confidence interval region, and the dotted eclipse represents the prediction region, a forecast of the true sensitivity and specificity in a future study. AUC: area under the receiver operating curve, Se: sensitivity, Sp: specificity.


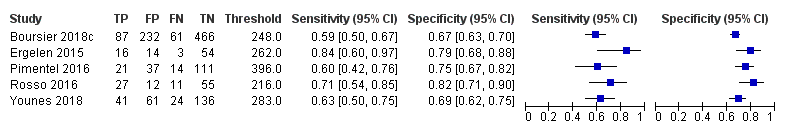


### S8 Fig. Forest plot of studies investigating diagnostic accuracy of CK-18 (M30) in detecting advanced fibrosis. TP: true positive, FP: false positive, FN: false negative, TN: true negative.
